# Dialysis session timing and outcomes: mortality and hospitalization differences across morning, afternoon, and night shifts in hemodialysis patients

**DOI:** 10.1080/0886022X.2025.2568648

**Published:** 2025-10-06

**Authors:** Xindong Wu, Weidong Zhang, Xiaoyan Jiao, Jialin Wang, Zhonghua Liu, Xuesen Cao, Fangfang Xiang, Yang Li, Bo Shen, Jianzhou Zou, Yongmei Zhang, Xiaoqiang Ding, Jieru Cai

**Affiliations:** a Department of Nephrology, Zhongshan Hospital, Fudan University, Shanghai, China; b Blood Purification Center, Zhongshan Hospital, Fudan University, Shanghai, China

**Keywords:** Dialysis session timing, hemodialysis, mortality, hospitalization, circadian variation

## Abstract

The study aims to investigate the association between dialysis shift and all-cause death and hospitalization among patients on hemodialysis (HD). In this single-center retrospective analysis, we enrolled 395 patients on HD who received treatment at our center on June 1, 2022, with a 2-year follow-up period. Participants were grouped into three dialysis shifts: morning shift, afternoon shift, and night shift. The primary outcome was all-cause mortality. The secondary outcome was hospitalizations and the association between hospitalization and clinical parameters. A total of 395 patients were analyzed for all-cause mortality. Kaplan–Meier analysis revealed a significantly elevated mortality among afternoon-shift patients compared to other shift groups (*p* = 0.013). Multivariable Cox regression confirmed that afternoon-shift was independently associated with an increased risk of mortality (adjusted HR 1.697, 95% CI 1.028–2.804). During a follow-up of two years, the remaining 272 surviving patients were evaluated for hospitalization events. The total number of hospitalization events and hospitalization per person-year were significantly lower in the night-shift group compared to other shifts. Furthermore, this group demonstrated the lowest incidence of access-related/HD-related hospitalization. Subsequent analyses identified: (1) negative association between SpKt/V and non-access-related events; (2) negative association between serum calcium and all-cause hospitalization; (3) negative correlation between left ventricular ejection fraction and access-related/HD-related events. Dialysis shift is associated with all-cause mortality and hospitalizations among patients on HD. However, this relationship is not directly driven by the temporal effects of the shift but rather by the fact that individuals with similar clinical characteristics tend to choose the same shift, leading to shift-specific differences in health outcomes.

## Graphical Abstract

**Figure:**
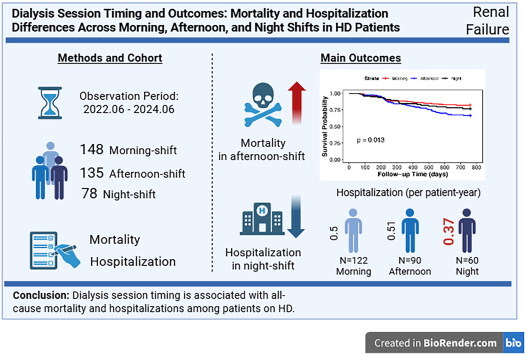

## Introduction

As a global public health crisis, end-stage kidney disease (ESKD) is a leading cause of morbidity and mortality worldwide [1,2]. Renal replacement therapy is the primary treatment for these patients, with hemodialysis (HD) and peritoneal dialysis (PD) being the most common modalities [1]. Unlike PD, which can be performed at home, in China, HD is routinely provided at a dialysis center [2]. Due to equipment limitations, short treatment duration, and safety considerations, dialysis centers typically adopt a multi-shift schedule [3,4], assigning patients to a specific shift based on their preferences and the unit available.

Circadian biology is well documented to regulate critical functions such as hormone secretion, sleep-wake cycles, and cardiovascular activity [5–9]. As a potent physiological intervention, the timing of dialysis likely disrupts these circadian processes. Preliminary research suggests that dialysis shift timing may significantly impact quality of life, affecting both sleep patterns and mood in the HD population, although these findings remain inconsistent [3,4,10,11].

Crucially, the association between dialysis shift timing and major clinical outcomes—specifically, all-cause mortality and hospitalization—has not been well characterized among patients on HD, despite this population bearing an estimated 6.5 times higher risk of all-cause mortality and 3-fold greater hospitalization probability compared with the general population [12,13]. This study examines the association between dialysis shift timing and these critical outcomes, aiming to provide evidence to guide optimal shift allocation strategies and potentially improve patient prognosis.

## Materials and methods

### Participants

**Figure 1.**
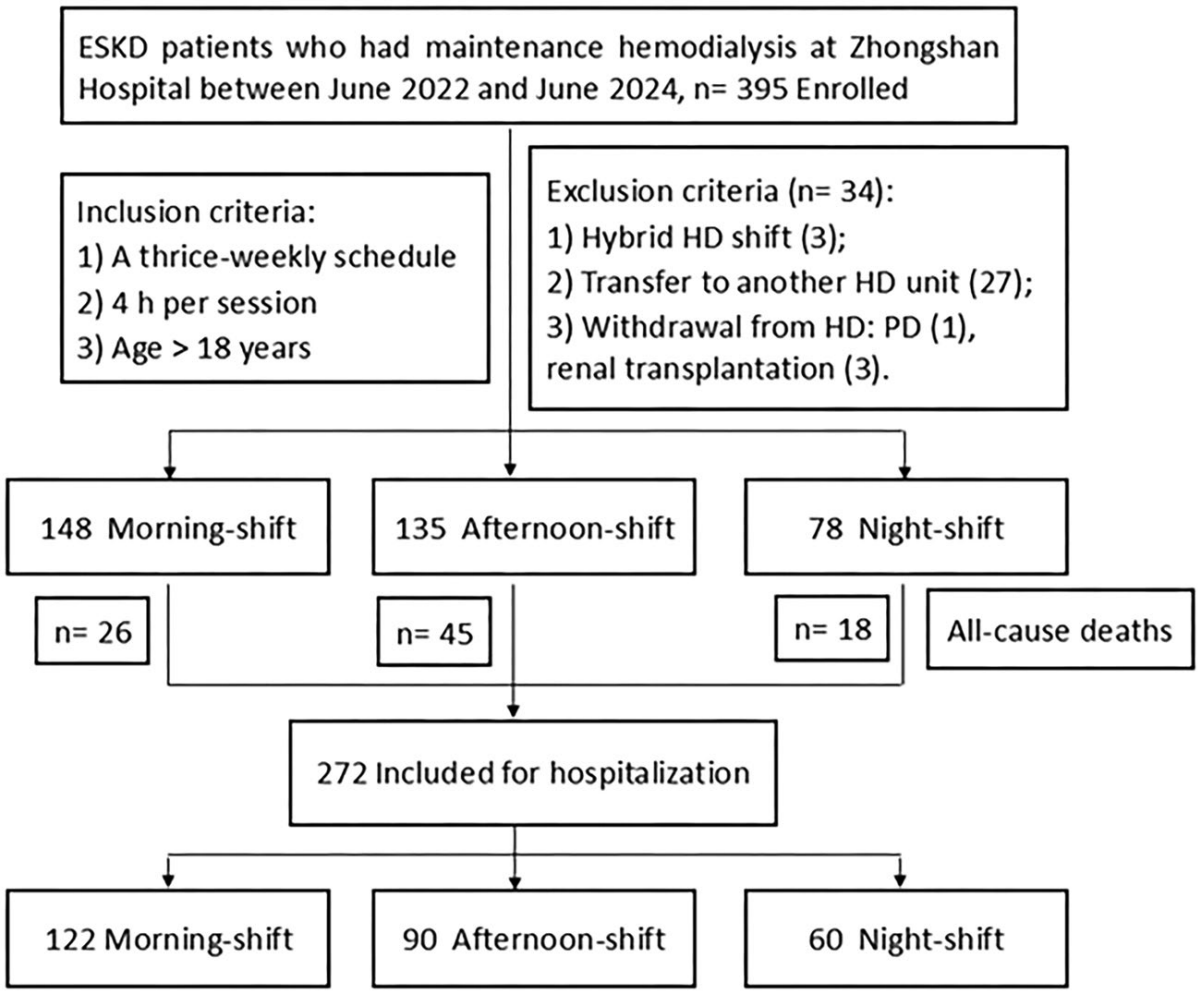
Flow chart. ESKD, end-stage kidney disease; HD, hemodialysis; PD, peritoneal dialysis. The definition of *hybrid shifts* cases: irregular shift patterns (e.g. alternating morning/afternoon/night sessions weekly).

The retrospective analysis was conducted with approval from the Ethical Committee of Zhongshan Hospital, Fudan University (Approval Number: Y2023-599). 395 ESKD patients were enrolled who had maintenance hemodialysis (MHD) at the Blood Purification Center, Department of Nephrology, Zhongshan Hospital, Fudan University on June 1, 2022, with a 2-year follow-up period from June 2022 to June 2024. All subjects provided written informed consent. The inclusion criteria were: 1) on a thrice-weekly schedule and 4-h per session; 2) age > 18 years. Exclusion criteria were (*n* = 34): 1) hybrid dialysis shift (in irregular shift pattern during observation period), *n* = 3; 2) transfer to another HD unit, *n* = 27; 3) withdrawal from HD, *n* = 4: renal transplantation, PD. (Figure 1)

### HD scheduling allocation

Our HD center runs three sessions per day. Based on the scheduled treatment times, these are categorized as: morning-shift (7:30 − 12:30), afternoon-shift (13:00–18:00), night-shift (18:30 − 23:30). The assignment of HD scheduling should be determined through an integrated evaluation of patient preferences, slot availability, and individual clinical status. A 1:4–5 nurse-to-machine ratio was maintained for all HD shifts.

### Data collection

Demographic characteristics, medical history, and laboratory measurements were extracted from the electronic health records system. All laboratory parameters were performed at baseline (study initiation).

The spKt/V was calculated according to the KDOQI Guidelines (2015) using the following formula [14]: −ln(*R* − 0.008t) + (4 − 3.5 R) × ΔBW/BW, where R = post-HD BUN/pre-HD BUN, t = dialysis time (hours), ΔBW = ultrafiltration volume, BW = post-HD weight.

### Outcomes

The primary outcome was all-cause mortality. The secondary outcome were hospitalizations, which were classified into six categories based on etiology: (1) access dysfunction; (2) access-related infection; (3) fluid overload; (4) non-access-related infection (e.g. pneumonia, gastrointestinal infection); (5) cardiovascular events (e.g. myocardial infarction, stroke, epilepsy); (6) other causes (e.g. gynecological surgery, cataract surgery, endoscopic procedures). For analytical purposes, hospitalizations were further categorized as access-related (categories 1–2) vs. non-access related (categories 3–6); HD-related (categories 1–3) vs. non-HD-related (categories 4–6). The study also evaluated the association between hospitalization and clinical parameters.

### Statistics analysis

Quantitative data that followed a normal distribution were presented as means ± standard deviations (SDs) and compared among multiple groups using one-way ANOVA. Non-normally distributed quantitative variables were expressed as median [interquartile range (IQR)] and analyzed using the Kruskal-Wallis test. Hospitalization data were categorized as categorical variables, reported as the number of cases [percentages (%)], and a chi-square test was performed for group comparisons.

Cumulative mortality rates were evaluated with the Kaplan-Meier method, and group differences were assessed by the log-rank test. The association between dialysis shift and mortality was studies used Cox proportional hazards regression, with results reported as hazard ratios (HRs) and 95% confidence intervals (CIs). Both univariate and multivariate Cox models were employed. The selection of variables for the multivariate model was informed by the univariate regression results. Spearman’s rank-order correlation analysis assessed relationships between clinical parameters and hospitalization frequency.

All statistical analysis was performed using the STATA statistical software program (STATA version 17.0; Stata Corp, College Station, TX). Differences with *P* values < 0.05 were considered statistically significant.

## Results

### Patients characteristics

395 patients were enrolled to evaluate all-cause mortality. During the 24-month observation period, 89 deaths occurred (26 morning-shift, 45 afternoon-shift, 18 night-shift), and the 272 survivors were analyzed for hospitalization (Figure 1).

Significant intergroup differences were observed in age, dialysis vintage, and employment rate. Night-shift patients were the youngest (*p* < 0.001), had the shortest dialysis vintage (*p* < 0.001), and had the highest employment rate (*p* = 0.010). However, these patients also exhibited a significantly higher prevalence of diabetes (*p* = 0.033) and a high proportion of CVC use (*p* = 0.017). Morning-shift patients exhibited lower pro-BNP levels (*p* = 0.021). No significant differences were observed among groups in other parameters, including dialysis adequacy (SpKt/V, *p* = 0.140) and cardiac function (LVEF, *p* = 0.807) (Table 1).

**Table 1.** Baseline demographic and clinical characteristics by dialysis shift.

Factor	Morning (*n* = 148)	Afternoon (*n* = 135)	Night (*n* = 78)	*P*
1. Baseline characteristics				
Age, yr	59.57 (49.00, 70.00)	65.33 (56.00, 73.00)	58.18 (47.75, 67.25)	*<0.001*
Male, n (%)	87 (58.8)	91 (67.4)	49 (62.8)	0.325
BMI, n (%)				
<18	27 (18.2)	28 (20.7)	10 (12.8)	0.450
18.5-23.9	80 (54.1)	68 (50.4)	40 (51.3)	
24-27.9	25 (16.9)	29 (21.5)	16 (20.5)	
≥28	16 (10.8)	10 (7.4)	12 (15.4)	
Marital status				
Single, n (%)	28 (18.9)	10 (7.4)	9 (11.5)	0.059
Married, n (%)	106 (71.6)	108 (80.0)	61 (78.2)	
Divorced, n (%)	8 (5.4)	5 (3.7)	1 (1.3)	
Widowed, n (%)	6 (4.1)	12 (8.9)	7 (9.0)	
Educational level				
Primary and below, n (%)	30 (20.3)	22 (16.3)	14 (17.9)	0.365
High school, n (%)	80 (54.1)	84 (62.2)	39 (50.0)	
College and above, n (%)	38 (25.7)	29 (21.5)	25 (32.1)	
Employed, n (%)	12 (8.1)	5 (3.7)	12 (15.4)	*0.010*
HBV/HCV positive, n (%)	13 (8.8)	13 (9.6)	6 (7.7)	0.891
2. Comorbidity				
Hypertension, n (%)	126 (85.1)	122 (90.4)	68 (87.2)	0.410
Diabetes, n (%)	30 (20.3)	32 (23.7)	28 (35.9)	*0.033*
Dyslipidemia, n (%)	32 (21.6)	25 (18.5)	22 (28.2)	0.256
CAD, n (%)	49 (33.1)	44 (32.6)	28 (35.9)	0.878
3. Dialysis-associated data				
CVC, n (%)	28 (18.9)	24 (17.8)	26 (33.3)	*0.017*
Vintage, yrs	9.62 ± 5.98	9.22 ± 5.83	6.53 ± 3.93	*<0.001*
Interdialytic weight gain, %	3.90 (3.00, 4.60)	3.75 (2.90, 4.40)	3.95 (3.40, 4.50)	0.238
SpKt/V	1.23 (1.11, 1.36)	1.26 (1.11, 1.36)	1.19 (1.07, 1.30)	0.140
Hb, g/L	106.59 ± 15.26	105.81 ± 16.79	106.15 ± 17.90	0.928
Alb, g/L	41.17 (39.00, 44.00)	40.86 (38.00, 43.00)	40.79 (39.00, 43.00)	0.541
Ca, mmol/L	2.28 ± 0.23	2.32 ± 0.29	2.27 ± 0.26	0.264
P, mmol/L	2.36 ± 0.68	2.18 ± 0.62	2.26 ± 0.64	0.068
PTH, pg/mL	2.32 ± 0.43	2.40 ± 0.37	2.36 ± 0.38	0.302
pro-BNP, pg/mL	3.71 ± 0.54	3.87 ± 0.51	3.86 ± 0.58	*0.021*
LVEF, %	60.43 (59.00, 66.75)	60.13 (59.00, 67.00)	60.51 (59.00, 65.00)	0.807

PTH and pro-BNP values were logarithmically transformed (base 10) prior to statistical analysis.

Abbreviation: BMI, body mass index; HBV/HCV, hepatitis B/hepatitis C; CAD, coronary artery disease; CVC, central venous catheter; spKt/V, single-pool Kt/V; Hb, hemoglobin; Alb, serum albumin; Ca, serum calcium; P, serum phosphate; PTH, parathyroid hormone; pro-BNP, N-terminal pro-B-type natriuretic peptide; EF%, left ventricular ejection fraction.

### All-cause mortality and hospitalization patterns across dialysis shift

**Figure 2.**
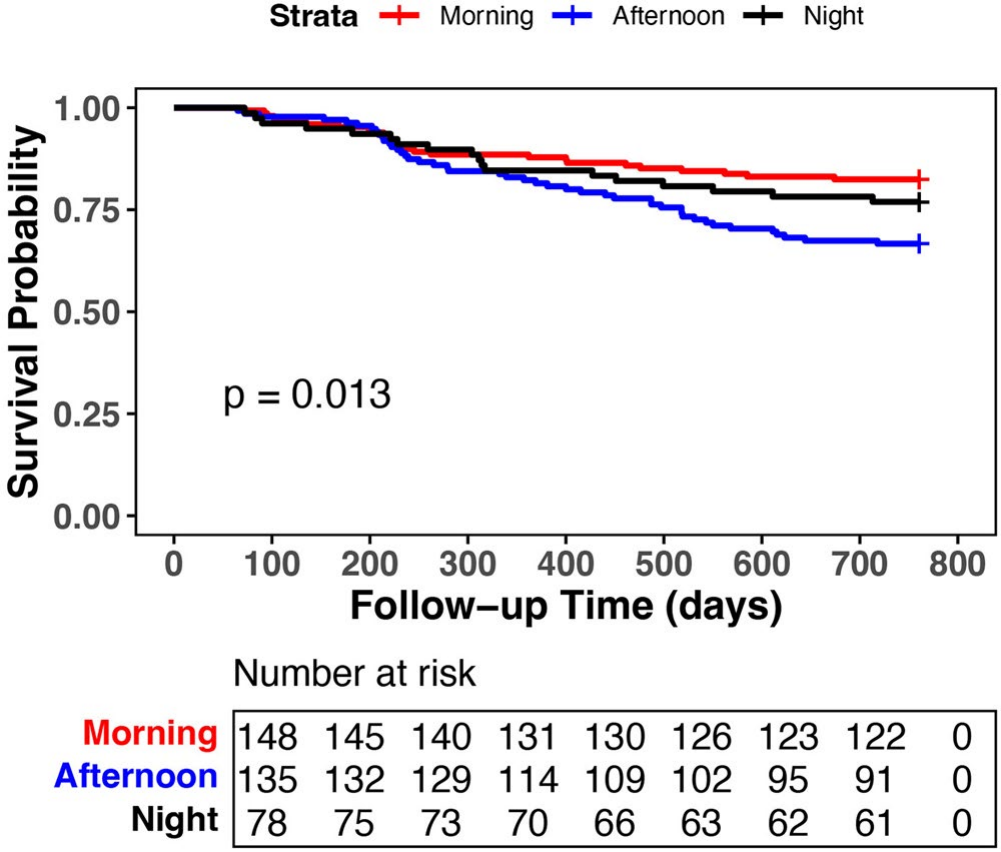
Kaplan–Meier Curves for all-cause mortality by dialysis shift.

As shown in Figure 2, Kaplan–Meier analysis revealed a significantly elevated mortality among afternoon-shift patients compared to other shift groups (*p* = 0.013), with deceased patients in this group also being the oldest (the mean age 67.12 ± 13.17 years vs. 60.51 ± 17.21 morning and 61.05 ± 13.62 night; *p* = 0.032). Multivariable Cox regression, adjusted for potential confounding factors listed in Table 2, confirmed that the afternoon-shift was independently associated with an increased risk of mortality (adjusted HR 1.697, 95% CI 1.028–2.804). Subgroup analysis revealed that patients in the afternoon-shift had a significantly higher mortality rate compared to other shifts among those who underwent dialysis during the non-COVID-19 peak period (adjusted HR 2.462, 95% CI 1.108–5.471) and those with an arteriovenous fistula access (adjusted HR 1.937, 95% CI 1.068–3.515) (Table S1-S3).

**Table 2.** Cox proportional hazards regression analysis of all-cause mortality by dialysis shift.

	Univariable Cox Regression Analysis		Multivariable Cox Regression Analysis	
	HR (95% CI)	*P*	HR (95% CI)	*P*
Dialysis shift				
Morning	Ref		Ref	
Afternoon	2.015 (1.243, 3.267)	*0.004*	1.697 (1.028, 2.804)	*0.039*
Night	1.341 (0.735, 2.447)	0.338	1.067 (0.571, 1.994)	0.839
Age	1.018 (1.002, 1.035)	*0.032*	1.005 (0.987, 1.024)	0.567
Sex				
Male	Ref		Ref	
Female	0.844 (0.544, 1.310)	0.450	0.893 (0.559, 1.426)	0.634
Diabetes	1.613 (1.035, 2.513)	*0.035*	1.289 (0.798, 2.081)	0.300
LVEF	0.978 (0.960, 0.996)	*0.018*	1.000 (0.980, 1.022)	0.966
pro-BNP	3.447 (2.235, 5.315)	*<0.001*	2.561 (1.552, 4.225)	*<0.001*
Access				
AVF	Ref		Ref	
CVC	1.817 (1.162, 2.843)	*0.009*	1.209 (0.750, 1.950)	0.436
Employment	0.505 (0.185, 1.378)	0.182	0.916 (0.296, 2.828)	0.878
Education				
Primary and below	Ref		Ref	
High school	0.730 (0.427, 1.248)	0.250	0.986 (0.564, 1.723)	0.960
College and above	0.922 (0.508, 1.674)	0.789	1.050 (0.560, 1.969)	0.878

Abbreviation: pro-BNP, N-terminal pro-B-type natriuretic peptide; LVEF%, left ventricular ejection fraction; AVF, arteriovenous fistula; CVC, central venous catheter.

Among surviving patients during the observation period, night-shift patients had the lowest total hospitalization events (44 vs. 122 morning and 92 afternoon) and hospitalization frequency (0.37 per person-year vs. 0.50 morning and 0.51 afternoon). When stratified by etiology, hospitalization patterns varied significantly across shifts (Table 3). Both morning- and afternoon-shift patients experienced higher total events and frequency of access-related hospitalizations than night-shift patients (*p* = 0.025). A similar trend was observed in HD-related hospitalization events (*p* = 0.044).

**Table 3.** Patterns of hospitalization across dialysis shift.

Variables	Morning (*n* = 122)	Afternoon (*N* = 90)	Night (*N* = 60)	*P*
Total Hospitalization Events	122	92	44	–
Hospitalization per patient-year	0.50	0.51	0.37	–
Etiology of Hospital Admission				
access dysfunction	47 (18.2)	43 (16.7)	9 (3.5)	*<0.001*
access related infection	4 (1.6)	0	1 (0.4)	
fluid overload	16 (6.2)	11 (4.3)	6 (2.3)	
non-access related infection	16 (6.2)	15 (5.8)	4 (1.6)	
cardiovascular events	0	4 (1.6)	5 (1.9)	
other	39 (15.1)	19 (7.4)	19 (7.4)	
Cause whether based on access				
access-related	51 (41.8)	43 (46.7)	10 (22.7)	*0.025*
non-access-related	71 (58.2)	49 (53.3)	34 (77.3)	
Cause whether related with HD				
HD-related	67 (54.9)	54 (58.7)	16 (36.4)	*0.044*
non-HD related	55 (45.1)	38 (41.3)	28 (63.6)	

### The relationship between clinical parameters and hospitalization patterns

As demonstrated in Table 4, significant associations were observed between clinical parameters and hospitalization patterns, with adjustment for age, sex, BMI, dialysis vintage, employment and education level as covariates. Dialysis adequacy (SpKt/V) was inversely correlated with non-access-related hospitalization events (*p* = 0.025). Serum calcium level showed a significant negative correlation with all-cause hospitalization event (*p* < 0.05), though its association with access-related events did not reach statistical significance. Reduced LVEF was negatively correlated with higher access-related (*r*= −0.147, *p* = 0.016) and HD-related events (*r*= −0.162, *p* = 0.006).

**Table 4.** The relationship between clinical parameters and hospitalization patterns.

Variables	Access-related			Non-access-related			HD-related			Non-HD-related		
	*r*	95% CI	*P*	*r*	95% CI	*P*	*r*	95% CI	*P*	*r*	95% CI	*P*
SpKt/V	0.072	(−0.020, 0.169)	0.244	−0.138	(−0.270, 0.001)	*0.024*	−0.005	(−0.129, 0.107)	0.935	−0.106	(−0.233, 0.024)	0.083
Hb, g/L	0.000	(−0.120, 0.099)	1.000	−0.037	(−0.166, 0.085)	0.544	0.013	(−0.095, 0.115)	0.836	−0.061	(−0.181, 0.064)	0.323
Alb, g/dL	−0.071	(−0.202, 0.056)	0.246	−0.027	(−0.183, 0.109)	0.663	−0.087	(−0.205, 0.045)	0.159	−0.007	(−0.170, 0.129)	0.903
Ca, mmol/L	−0.100	(−0.200, −0.010)	0.104	−0.147	(−0.279, −0.029)	*0.017*	−0.123	(−0.218, −0.29)	*0.045*	−0.148	(−0.267, −0.039)	*0.016*
P, mg/dL	−0.011	(−0.126, 0.123)	0.860	−0.051	(−0.198, 0.132)	0.403	−0.024	(−0.164, 0.131)	0.700	−0.050	(−0.190, 0.128)	0.413
PTH, pg/mL	0.018	(−0.135, 0.157)	0.774	0.020	(−0.106, 0.119)	0.742	0.038	(−0.110, 0.170)	0.539	0.002	(−0.129, 0.112)	0.972
pro-BNP, pg/mL	0.079	(−0.024, 0.191)	0.201	0.062	(−0.046, 0.197)	0.316	0.056	(−0.040, 0.162)	0.365	0.091	(−0.029, 0.227)	0.140
LVEF, %	−0.168	(−0.303, −0.050)	*0.006*	−0.072	(−0.189, 0.057)	0.239	−0.184	(−0.307, −0.066)	*0.003*	−0.050	(−0.167, 0.094)	0.413

PTH and pro-BNP values were logarithmically transformed (base 10) prior to statistical analysis.

Abbreviation: HD, hemodialysis; spKt/V, single-pool Kt/V; Hb, hemoglobin; Alb, serum albumin; Ca, serum calcium; P, serum phosphate; PTH, parathyroid hormone; pro-BNP, N-terminal pro-B-type natriuretic peptide; LVEF, left ventricular ejection fraction.

## Discussion

In this study, we found dialysis scheduling-based variations in hemodialysis health outcomes: (1) peak all-cause mortality in the afternoon-shift, (2) lowest hospitalization events and frequency in the night-shift, and (3) elevated proportion of access/HD-related hospitalizations in the morning- and afternoon-shifts.

Our study investigated whether mortality was associated with dialysis shift schedules. Notably, we found that patients in the afternoon-shift exhibited the highest all-cause mortality across all shifts, with the deceased patients in this group also being the oldest at the time of death. The K-M curve revealed a pronounced increase in mortality risk, evidenced by *a* ≈ 50% steeper decline in survival probability between June 2022 and March 2023. The temporal association coincided with China’s peak phase of the COVID-19 surge. Salerno S et al. established that COVID-19 may contribute to mortality in patients on HD [15]. Due to a lack of COVID-19 status for all deceased patients, whether the COVID-19 pandemic likely amplified baseline shift-related mortality needs to be further studied. However, subgroup analysis of the period after the COVID-19 pandemic (i.e. between April 2023 and June 2024) revealed that mortality remained highest in afternoon-shift. This finding further supports the inherent risks associated with the afternoon-shift.

Despite advances in HD delivery, patients with ESKD remain at high cardiovascular risk [16]. While Elliot WJ et al. demonstrated that cardiovascular disease has an intrinsic events variation and morning surges in cardiovascular risk are widely documented in the general population [6]. Our hemodialysis cohort exhibited a reversed pattern: afternoon-shift patients showed both higher all-cause mortality and increased cardiovascular hospitalization events compared to morning-shift patients. Although the exact time of death was not recorded, afternoon dialysis is theoretically less disruptive to the circadian rhythm than dialysis performed during the morning or night shifts. These results suggest circadian variations alone don’t explain shift-related outcomes.

To ensure high-quality care, our dialysis center is equipped with sufficient medical resources and well-staffed medical teams. Night-shift patients showed decreasing overall hospitalization events, despite an increasing proportion of non-HD-related and non-access-related events. Our study found that those on the night-shift had a higher education level. As a result, they demonstrated stronger self-management capabilities in dialysis care and vascular access maintenance, leading to reduced hospitalization events due to HD-related or access-related events [17]. We also found that patients on the night-shift had shortest dialysis vintage. Dialysis vintage is associated with both increased mortality and physical function decline in patients on HD, which contributes to elevated hospitalization events [18–20]. Although dialysis vintage showed no significant association with mortality in adjusted Cox models (*p* = 0.087) in our analysis, the lower hospitalization observed in night-shift patients may still reflect benefits associated with their shorter vintage, warranting further investigation.

Additionally, some patients actively opted for night-dialysis to avoid work conflicts due to their higher employment rates [21]. The potential healthy worker effect (HWE), typically observed in occupational cohorts with baseline health advantage [22], warrants consideration in our study, particularly for night-shift patients with the highest employment rate. Patients in the night-shift were younger; however, this group’s clinical profile was inconsistent with conventional HWE assumptions: higher comorbidities burden (including diabetes, dyslipidemia, and CAD), and worse cardiac function. These findings challenged the presumption that employment indicates better health in dialysis populations. Patients on the afternoon-shift were more prone to access-related events, primarily attributed to the following factors: (1) older age and poorer baseline vascular conditions, (2) diminished self-care abilities due to age-related physical decline, and (3) vascular calcification exacerbating access deterioration [23–25]. In practice, the afternoon-shift is the preferred slot for many patients, particularly older individuals, as it aligns with their routine dietary and sleep schedules without significant disruption.

This study also found that hospitalization events were closely associated with the following factors. (1) Dialysis adequacy: there was a negative correlation between the dialysis adequacy (SpKt/V) and non-access-related events. Dialysis adequacy serves as a cornerstone, emphasizing that optimal dialysis is key to reducing complications [14]. (2) Cardiac function: a reduced LVEF was significantly associated with access-related/HD-related events, indicating that worsening cardiac function may increase the risk of systemic complications [26]. (3) Calcium level: there was a negative association between the serum calcium level and all-cause hospitalization. Maintaining calcium balance is critical for MHD patients as it significantly influences bone-mineral metabolism, cardiovascular function, and overall prognosis [27]. In our study, all enrolled patients exhibited comparable baseline serum calcium levels within the normal physiological range. The observed outcomes may not accurately reflect calcium-related pathological mechanisms. Together, adequate dialysis and improved cardiac function may help reduce hospitalization events.

Previous studies have indicated an association between dialysis shift schedules and patient depressive symptom as well as sleep quality [3,4, 10,11]. Although findings across studies are not entirely consistent, the temporal differences among shifts may influence these adverse outcomes. However, the impact of dialysis shifts on hospitalization events differs from these earlier observations. Shift timing does not directly affect hospitalization rates; rather, patients with distinct characteristics tend to self-select into specific shifts, leading to divergent risk profiles. For high-risk patients—such as those with poor cardiac function or advanced age—prioritizing morning-shifts is recommended. Beyond the dialysis center’s own resources, the broader healthcare system offers greater medical support during this time, enhancing emergency response capabilities and overall clinical outcomes [28–30].

There are several limitations that need to be addressed. First, as a single-center study, the generalizability of our findings may be constrained. Future multicenter prospective studies or registry-based designs with larger, more diverse patient cohorts are warranted to validate the observation and provide stronger evidence to confirm these associations beyond local practice patterns. Second, although we excluded patients with hybrid dialysis shifts at baseline, some individuals experienced temporary shift adjustments or suspensions during the 2-year observation period, which may have introduced bias to the results. Third, the COVID-19 pandemic during the study period likely increased all-cause mortality among the cohort, potentially obscuring the true association between dialysis shift and mortality risk.

## Conclusion

Dialysis shift schedule is associated with all-cause mortality and hospitalizations among patients on HD. However, this relationship appears to be primarily driven by inherent differences in patient characteristics across shifts rather than the temporal effects of the shifts themselves. These findings suggest that shift allocation strategies should account for patient-specific risk profiles rather than temporal factors alone.

## Supplementary Material

Supplement Figure 1 Graphical abstract R2.jpeg

Supplement Table 3 Cox Proportional Hazards Regression subgroup Access R2.docx

Supplement Table 2 Cox Proportional Hazards Regression subgroup Age R2.docx

Supplement Table 1 Cox Proportional Hazards Regression subgroup COVID R2.docx

## Data Availability

Data will be made available on request.
