# Supplementary figures and images for: Dialysis session timing and outcomes: mortality and hospitalization differences across morning, afternoon, and night shifts in hemodialysis patients
Source: Ren Fail. 2025 Oct 6;47(1):2568648. doi: 10.1080/0886022X.2025.2568648 (PMC12507099; doi:10.1080/0886022X.2025.2568648)

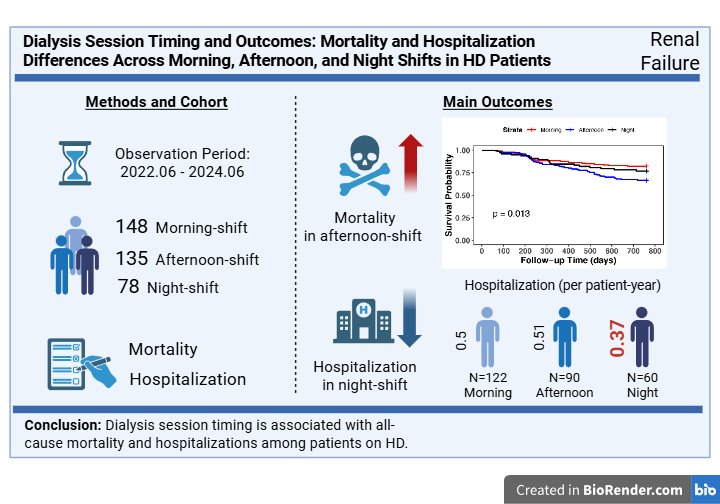

Supplement: Supplement Figure 1 Graphical abstract R2.jpeg [file IRNF_A_2568648_SM4884.jpeg]
