# Supplementary material for: Dialysis session timing and outcomes: mortality and hospitalization differences across morning, afternoon, and night shifts in hemodialysis patients
Source: Ren Fail. 2025 Oct 6;47(1):2568648. doi: 10.1080/0886022X.2025.2568648 (PMC12507099; doi:10.1080/0886022X.2025.2568648)
Supplement: Supplement Table 3 Cox Proportional Hazards Regression subgroup Access R2.docx [file IRNF_A_2568648_SM4883.docx]

| Table S3. Cox Proportional Hazards Regression Analysis of All-Cause Mortality by Dialysis Shift Stratified by Vascular Access Type. | | | | | | | | | | | |
| --- | --- | --- | --- | --- | --- | --- | --- | --- | --- | --- | --- |
|  | AVF | | | | |  | CVC | | | | |
|  | Univariable Cox Regression Analysis | |  | Multivariable Cox Regression Analysis | |  | Univariable Cox Regression Analysis | |  | Multivariable Cox Regression Analysis | |
|  | HR(95% CI) | *P* |  | HR(95% CI) | *P* |  | HR(95% CI) | *P* |  | HR(95% CI) | *P* |
| Dialysis shift |  |  |  |  |  |  |  |  |  |  |  |
| Morning | Ref |  |  | Ref |  |  | Ref |  |  | Ref |  |
| Afternoon | 2.165(1.222, 3.833) | ***0.008*** |  | 1.937(1.068, 3.515) | ***0.030*** |  | 1.718(0.690, 4.275) | 0.245 |  | 1.247(0.452, 3.440) | 0.670 |
| Night | 1.156(0.519, 2.573) | 0.722 |  | 0.931(0.414, 2.090) | 0.862 |  | 1.232(0.475, 3.194) | 0.668 |  | 1.610(0.531, 4.877) | 0.400 |
| Age | 1.004(0.983, 1.025) | 0.720 |  | 0.994(0.972, 1.018) | 0.629 |  | 1.028(1.004, 1.053) | ***0.022*** |  | 1.031(0.996, 1.067) | 0.085 |
| Sex |  |  |  |  |  |  |  |  |  |  |  |
| Male | Ref |  |  | Ref |  |  | Ref |  |  | Ref |  |
| Female | 0.570(0.318, 1.021) | 0.059 |  | 0.654(0.352, 1.216) | 0.179 |  | 1.582(0.752, 3.327) | 0.227 |  | 1.765(0.753, 4.138) | 0.191 |
| Diabetes | 1.774(1.032, 3.051) | ***0.038*** |  | 1.209(0.672, 2.173) | 0.527 |  | 1.084(0.500, 2.350) | 0.838 |  | 1.471(0.526, 4.116) | 0.462 |
| LVEF | 0.978(0.957, 1.000) | ***0.045*** |  | 1.006(0.981, 1.032) | 0.645 |  | 0.984(0.950, 1.020) | 0.386 |  | 0.972(0.927, 1.019) | 0.241 |
| pro-BNP | 3.406(2.038, 5.691) | ***<0.001*** |  | 2.409(1.302, 4.457) | ***0.005*** |  | 2.798(1.203, 6.507) | ***0.017*** |  | 2.613(0.995, 6.682) | 0.051 |
| Employment | 0.638(0.200, 2.038) | 0.449 |  | 1.147(0.328, 4.007) | 0.830 |  | 0.259(0.035, 1.906) | 0.185 |  | 0.642(0.057, 7.251) | 0.720 |
| Education |  |  |  |  |  |  |  |  |  |  |  |
| Primary and below | Ref |  |  | Ref |  |  | Ref |  |  | Ref |  |
| High school | 0.591(0.312, 1.118) | 0.106 |  | 0.827(0.415, 1.648) | 0.589 |  | 1.291(0.473, 3.525) | 0.618 |  | 1.391(0.457, 4.232) | 0.561 |
| College and above | 0.869(0.432, 1.747) | 0.694 |  | 0.960(0.453, 2.035) | 0.916 |  | 1.094(0.347, 3.448) | 0.878 |  | 1.536(0.435, 5.421) | 0.504 |

Abbreviation: pro-BNP, N-terminal pro-B-type natriuretic peptide; LVEF%, left ventricular ejection fraction; AVF, arteriovenous fistula; CVC, central venous catheter.
