# Supplementary material for: Dialysis session timing and outcomes: mortality and hospitalization differences across morning, afternoon, and night shifts in hemodialysis patients
Source: Ren Fail. 2025 Oct 6;47(1):2568648. doi: 10.1080/0886022X.2025.2568648 (PMC12507099; doi:10.1080/0886022X.2025.2568648)
Supplement: Supplement Table 2 Cox Proportional Hazards Regression subgroup Age R2.docx [file IRNF_A_2568648_SM4882.docx]

| Table S2. Cox Proportional Hazards Regression Analysis of All-Cause Mortality by Dialysis Shift Stratified by Age. | | | | | | | | | | | |
| --- | --- | --- | --- | --- | --- | --- | --- | --- | --- | --- | --- |
|  | Younger (≤65) | | | | |  | Older (>65) | | | | |
|  | Univariable Cox Regression Analysis | |  | Multivariable Cox Regression Analysis | |  | Univariable Cox Regression Analysis | |  | Multivariable Cox Regression Analysis | |
|  | HR(95% CI) | *P* |  | HR(95% CI) | *P* |  | HR(95% CI) | *P* |  | HR(95% CI) | *P* |
| Dialysis shift |  |  |  |  |  |  |  |  |  |  |  |
| Morning | Ref |  |  | Ref |  |  | Ref |  |  | Ref |  |
| Afternoon | 2.066(1.041, 4.100) | ***0.038*** |  | 1.696(0.800, 3.597) | 0.168 |  | 1.870(0.928, 3.771) | 0.080 |  | 1.748(0.863, 3.539) | 0.121 |
| Night | 1.077(0.471, 2.460) | 0.861 |  | 0.957(0.387, 2.368) | 0.924 |  | 1.821(0.754, 4.394) | 0.183 |  | 1.384(0.553, 3.461) | 0.488 |
| Sex |  |  |  |  |  |  |  |  |  |  |  |
| Male | Ref |  |  | Ref |  |  | Ref |  |  | Ref |  |
| Female | 0.764(0.384, 1.520) | 0.443 |  | 0.987(0.464, 2.101) | 0.973 |  | 0.833(0.465, 1.492) | 0.539 |  | 0.841(0.451, 1.569) | 0.587 |
| Diabetes | 2.124(1.139, 3.960) | ***0.018*** |  | 1.412(0.689, 2.894) | 0.346 |  | 1.213(0.640, 2.299) | 0.553 |  | 1.176(0.586, 2.359) | 0.648 |
| LVEF | 0.972(0.949, 0.996) | ***0.023*** |  | 0.995(0.968, 1.022) | 0.702 |  | 0.980(0.952, 1.009) | 0.177 |  | 1.008(0.972, 1.046) | 0.656 |
| pro-BNP | 4.029(2.174, 7.465) | ***<0.001*** |  | 2.678(1.293, 5.550) | ***0.008*** |  | 3.028(1.584, 5.791) | ***<0.001*** |  | 2.368(1.157, 4.843) | ***0.018*** |
| Access |  |  |  |  |  |  |  |  |  |  |  |
| AVF | Ref |  |  | Ref |  |  | Ref |  |  | Ref |  |
| CVC | 1.216(0.582, 2.541) | 0.603 |  | 0.951(0.425, 2.127) | 0.903 |  | 2.365(1.320, 4.237) | ***0.004*** |  | 1.565(0.838, 2.925) | 0.160 |
| Employment | 0.609(0.217, 1.707) | 0.609 |  | 0.976(0.310, 3.077) | 0.967 |  | - | - |  | - | - |
| Education |  |  |  |  |  |  |  |  |  |  |  |
| Primary and below | Ref |  |  | Ref |  |  | Ref |  |  | Ref |  |
| High school | 0.530(0.250, 1.127) | 0.099 |  | 0.686(0.292, 1.612) | 0.387 |  | 1.029(0.478, 2.213) | 0.943 |  | 1.492(0.683, 3.258) | 0.316 |
| College and above | 0.567(0.241, 1.334) | 0.194 |  | 0.805(0.305, 2.128) | 0.662 |  | 1.592(0.689, 3.680) | 0.276 |  | 1.432(0.589, 3.484) | 0.429 |

Abbreviation: pro-BNP, N-terminal pro-B-type natriuretic peptide; LVEF, left ventricular ejection fraction; AVF, arteriovenous fistula; CVC, central venous catheter.
