# Supplementary material for: Dialysis session timing and outcomes: mortality and hospitalization differences across morning, afternoon, and night shifts in hemodialysis patients
Source: Ren Fail. 2025 Oct 6;47(1):2568648. doi: 10.1080/0886022X.2025.2568648 (PMC12507099; doi:10.1080/0886022X.2025.2568648)
Supplement: Supplement Table 1 Cox Proportional Hazards Regression subgroup COVID R2.docx [file IRNF_A_2568648_SM4881.docx]

| Table S1. Cox Proportional Hazards Regression Analysis of All-Cause Mortality by Dialysis Shift Stratified by Period. | | | | | | | | | | | |
| --- | --- | --- | --- | --- | --- | --- | --- | --- | --- | --- | --- |
|  | 2022.06.01-2023.03.31 | | | | |  | 2023.04.01-2024.06.30 | | | | |
|  | Univariable Cox Regression Analysis | |  | Multivariable Cox Regression Analysis | |  | Univariable Cox Regression Analysis | |  | Multivariable Cox Regression Analysis | |
|  | HR(95% CI) | *P* |  | HR(95% CI) | *P* |  | HR(95% CI) | *P* |  | HR(95% CI) | *P* |
| Dialysis shift |  |  |  |  |  |  |  |  |  |  |  |
| Morning | Ref |  |  | Ref |  |  | Ref |  |  | Ref |  |
| Afternoon | 1.364(0.720, 2.585) | 0.341 |  | 1.227(0.631, 2.386) | 0.547 |  | 3.286(1.527, 7.071) | ***0.002*** |  | 2.462(1.108, 5.471) | ***0.027*** |
| Night | 0.893(0.386, 2.070) | 0.792 |  | 0.683(0.283, 1.644) | 0.394 |  | 2.199(0.893, 5.411) | 0.086 |  | 1.987(0.780, 5.061) | 0.150 |
| Age | 1.008(0.986, 1.030) | 0.502 |  | 0.990(0.966, 1.015) | 0.439 |  | 1.030(1.005, 1.055) | ***0.016*** |  | 1.025(0.997, 1.054) | 0.086 |
| Sex |  |  |  |  |  |  |  |  |  |  |  |
| Male | Ref |  |  | Ref |  |  | Ref |  |  | Ref |  |
| Female | 0.802(0.433, 1.486) | 0.483 |  | 0.877(0.449, 1.712) | 0.700 |  | 0.891(0.476, 1.668) | 0.717 |  | 0.896(0.460, 1.748) | 0.748 |
| Diabetes | 2.295(1.276, 4.127) | ***0.006*** |  | 2.020(1.054, 3.872) | ***0.034*** |  | 1.037(0.511, 2.103) | 0.920 |  | 0.717(0.335, 1.533) | 0.391 |
| LVEF | 0.986(0.960, 1.012) | 0.291 |  | 1.005(0.976, 1.034) | 0.760 |  | 0.971(0.946, 0.995) | ***0.020*** |  | 0.992(0.961, 1.023) | 0.603 |
| pro-BNP | 3.426(1.868, 6.283) | ***<0.001*** |  | 2.094(1.065, 4.118) | ***0.032*** |  | 3.468(1.869, 6.437) | ***<0.001*** |  | 3.088(1.461, 6.527) | ***0.003*** |
| Access |  |  |  |  |  |  |  |  |  |  |  |
| AVF | Ref |  |  | Ref |  |  | Ref |  |  | Ref |  |
| CVC | 2.007(1.094, 3.682) | ***0.024*** |  | 1.393(0.726, 2.674) | 0.319 |  | 1.620(0.832, 3.154) | 0.156 |  | 1.076(0.530, 2.188) | 0.839 |
| Employment | 0.511(0.124, 2.108) | 0.353 |  | 0.883(0.182, 4.295) | 0.878 |  | 0.500(0.121, 2.067) | 0.338 |  | 1.079(0.213, 5.469) | 0.927 |
| Education |  |  |  |  |  |  |  |  |  |  |  |
| Primary and below | Ref |  |  | Ref |  |  | Ref |  |  | Ref |  |
| High school | 0.586(0.290, 1.185) | 0.137 |  | 0.935(0.442, 1.980) | 0.861 |  | 0.973(0.418, 2.268) | 0.950 |  | 1.209(0.504, 2.899) | 0.671 |
| College and above | 0.711(0.319, 1.582) | 0.403 |  | 0.928(0.395, 2.181) | 0.863 |  | 1.280(0.511, 3.209) | 0.598 |  | 1.317(0.502, 3.458) | 0.576 |

Abbreviation: pro-BNP, N-terminal pro-B-type natriuretic peptide; LVEF%, left ventricular ejection fraction; AVF, arteriovenous fistula; CVC, central venous catheter.
